# PD-L1-armored CD19/CD22 dual-targeted CAR-T cell co-infusion bridging to allogeneic hematopoietic stem cell transplantation achieves 7-year sustained remission in an adult patient with early relapsed, chemorefractory B-cell acute lymphoblastic leukemia: a case report

**DOI:** 10.3389/fimmu.2026.1797562

**Published:** 2026-04-27

**Authors:** Huan Hua, Shupeng Wen, Xinzhi Han, Xuan Liu, Ziwei Zhou, Zhengrong Song, Xuejun Zhang, Fuxu Wang

**Affiliations:** 1 Department of Hematology, The Second Hospital of Hebei Medical University, Shijiazhuang, China; 2 Hebei Key Laboratory of Hematology, Shijiazhuang, China

**Keywords:** allogeneic hematopoietic stem cell transplantation, B-cell acute lymphoblastic leukemia, CD19, CD22, chimeric antigen receptor T cells, dual-targeted CAR-T, PD-L1 immune checkpoint

## Abstract

**Background:**

Antigen escape and PD-L1/PD-1 axis-mediated immunosuppression in the tumor microenvironment (TME) are the predominant drivers of treatment failure in adult patients with relapsed/refractory (R/R) B-cell acute lymphoblastic leukemia (B-ALL). CD19-targeted CAR-T monotherapy has limited ability to overcome these two core resistance mechanisms, and achieving long-term durable remission remains a critical unmet clinical need in adults with high-risk, chemorefractory B-ALL.

**Methods:**

This is a single-center, retrospective case report of an adult patient with early-relapsed, chemorefractory B-ALL. Two independent CAR-T products were manufactured from the patient’s autologous peripheral blood mononuclear cells (PBMCs): (1) CD19 CAR-T cells containing an anti-CD19 single-chain variable fragment (scFv), a CD28 transmembrane domain, a 4-1BB co-stimulatory domain, and a CD3ζ signaling domain; (2) PD-L1-armored CD22 CAR-T cells containing an anti-CD22 scFv, a CD8 transmembrane domain, a 4-1BB co-stimulatory domain, a CD3ζ signaling domain, and a membrane-tethered anti-PD-L1 scFv for spatially restricted immune checkpoint modulation. The two CAR-T products were co-infused at doses of 5.0×10^5^ cells/kg (CD19 CAR-T) and 3.1×10^5^ cells/kg (PD-L1-armored CD22 CAR-T), respectively. We evaluated the feasibility, anti-leukemic efficacy, and long-term safety profile of this regimen as a bridging strategy to allogeneic hematopoietic stem cell transplantation (allo-HSCT). This treatment was administered under an institutional compassionate use program approved by the Ethics Committee of the Second Hospital of Hebei Medical University (approval number: 2017-R207), with written informed consent obtained from the patient prior to all treatment procedures. Comprehensive diagnostic workup for B-ALL was performed using 8-color multiparameter flow cytometry (MFC), conventional G-banding cytogenetic analysis, and multiplex leukemia fusion gene screening. Serial lumbar punctures with triple intrathecal chemotherapy (dexamethasone 5 mg + methotrexate 10 mg + cytarabine 30 mg) were performed throughout the treatment course; no abnormalities were detected in cerebrospinal fluid (CSF) routine, biochemistry, or flow cytometry assays, and no evidence of central nervous system (CNS) leukemia involvement was observed at any time point. This study is a retrospective observational analysis of a single clinical case, not a prospective interventional clinical trial, and thus was exempt from clinical trial registration requirements in accordance with institutional and national regulatory guidelines for retrospective observational studies.

**Results:**

The patient achieved minimal residual disease (MRD)-negative complete remission (CR) on day 14 post-infusion, as confirmed by 8-color MFC (detection sensitivity: 0.01%) and next-generation sequencing (NGS) of immunoglobulin heavy chain (IGH) gene rearrangements (limit of detection [LOD]: 10^−6^). Peak *in vivo* expansion of CAR-T cells was observed on day 10 post-infusion, with CD19 CAR-T cells accounting for 43.25% and CD22 CAR-T cells accounting for 14.58% of circulating CD3^+^ T lymphocytes. Only grade 1 cytokine release syndrome (CRS), per the American Society for Transplantation and Cellular Therapy (ASTCT) consensus criteria, occurred and resolved completely with supportive care; no immune effector cell-associated neurotoxicity syndrome (ICANS) was observed. Following consolidative allo-HSCT, rapid hematopoietic reconstitution was achieved, with neutrophil engraftment on day +12 and platelet engraftment on day +14, consistent with the median engraftment timeline for haploidentical HSCT at our institution. Complete donor chimerism (99.86%) was confirmed by short tandem repeat (STR) analysis on day +30 post-transplantation. Notably, the patient maintained MRD-negative sustained remission for 7 consecutive years, with no occurrence of acute or chronic graft-versus-host disease (GVHD) or late treatment-related adverse events. Complete immune reconstitution was achieved by 24 months post-transplantation, with sustained functional immune recovery and a Functional Assessment of Cancer Therapy-Leukemia (FACT-Leu) total score of 158/172 at the 7-year follow-up.

**Conclusions:**

This case report details the clinical course of an adult patient with early-relapsed, chemorefractory B-ALL who achieved 7-year MRD-negative sustained remission after treatment with PD-L1-armored CD19/CD22 dual-targeted CAR-T cell co-infusion followed by consolidative allo-HSCT. Our preliminary clinical observation demonstrates that this integrated regimen may mitigate antigen escape and immunosuppressive TME-mediated drug resistance, and effectively function as a bridging strategy to allo-HSCT in high-risk patient populations. The 7-year event-free survival (EFS) and sustained disease control observed in this case provide valuable clinical insights for the structural optimization of armored CAR-T constructs and the design of future prospective clinical trials for R/R B-ALL.

## Introduction

Adult B-cell acute lymphoblastic leukemia (B-ALL) remains a formidable clinical challenge despite advances in multi-agent chemotherapy and molecularly targeted therapies. Approximately 30–60% of adult patients achieve long-term disease-free survival (DFS) with conventional first-line chemotherapy, yet more than 50% of patients eventually develop disease relapse, which is associated with a dismal prognosis (1). For patients with relapsed/refractory (R/R) B-ALL, salvage chemotherapy yields a second complete remission (CR2) in only 18–45% of cases, with a median overall survival (OS) of just 3–6 months in non-responders (2). Although allogeneic hematopoietic stem cell transplantation (allo-HSCT) represents the only potentially curative treatment modality for eligible patients who achieve CR2, fewer than 50% of relapsed adult B-ALL patients successfully reach this critical therapeutic milestone (3).

Chimeric antigen receptor T (CAR-T) cell therapy has revolutionized the treatment paradigm for R/R B-ALL. CD19-directed CAR-T cells have consistently yielded impressive overall response rates (ORRs) of 70–90% across multiple multicenter clinical trials (4, 5), with long-term follow-up data demonstrating sustained clinical benefit in a subset of responding patients. However, nearly 70% of adult patients who achieve CR after CD19 CAR-T therapy eventually relapse during long-term follow-up, particularly those with high baseline tumor burden and extramedullary disease (6). Antigen escape, predominantly driven by the loss or downregulation of the CD19 target antigen on leukemic blasts, is the primary mechanism of treatment failure following CD19 CAR-T therapy (7). Sotillo et al. elegantly demonstrated that acquired mutations and alternative splicing of CD19 confer resistance to CD19-directed CAR-T therapy (8), while other studies have identified a myeloid lineage switch as an alternative mechanism of relapse after CD19 CAR-T treatment (9).

CD22, a lineage-restricted B-cell antigen expressed in the vast majority of B-ALL cases with an expression profile similar to CD19, has emerged as a key alternative target to mitigate antigen escape (10). Fry et al. demonstrated that CD22-targeted CAR-T cells induced CR in 73% of patients with R/R B-ALL, including those who progressed after prior CD19-directed immunotherapy. However, antigen downregulation still drove disease relapse in most responding patients, highlighting the intrinsic limitations of single-target CAR-T monotherapy (11).

To overcome these challenges, dual-targeted CAR-T strategies that simultaneously target CD19 and CD22 have been developed as a promising approach to prevent antigen escape. Three main clinical strategies have been investigated for dual-targeted CAR-T therapy: (1) tandem CAR constructs incorporating two distinct antigen-binding domains into a single vector; (2) bicistronic vectors co-expressing two separate CAR molecules in a single T cell; and (3) co-infusion of two independently manufactured single-target CAR-T products (12). Preclinical studies by Qin et al. demonstrated that co-infusion of CD19 and CD22 CAR-T cells achieved superior anti-leukemic efficacy compared with sequential infusion of the two products. Notably, when both CAR constructs are co-transduced into the same T cell, co-transduction efficiency is limited to approximately 25%, a technical constraint that favors the co-infusion strategy, which enables independent optimization of each CAR product and flexible dose adjustment (13).

The immunosuppressive tumor microenvironment (TME) is another major barrier to durable CAR-T efficacy in B-ALL. The interaction between programmed cell death protein 1 (PD-1) and its ligand programmed death-ligand 1 (PD-L1) exerts a potent inhibitory effect on T-cell effector function, and PD-L1 expression is significantly upregulated in the leukemia microenvironment after CAR-T cell-mediated antigen encounter (14, 15). Preclinical studies have demonstrated that PD-1/PD-L1 pathway blockade protects CAR-T cells from activation-induced cell death, providing a strong rationale for integrating immune checkpoint inhibition directly into CAR-T constructs to enhance anti-tumor activity (16).

Given the high relapse rates after CAR-T monotherapy, consolidative allo-HSCT is widely recommended to achieve durable disease control in adult patients with R/R B-ALL (17). Jacoby et al. highlighted that allo-HSCT after CAR-T-induced CR can exert additional graft-versus-leukemia (GVL) effects and eliminate residual CAR-T-resistant leukemic clones (18). Recent clinical data from Molina et al. identified key factors associated with improved outcomes after post-CAR-T allo-HSCT, including pre-CAR-T disease status and baseline tumor burden (19). Accumulating clinical evidence has confirmed that CAR-T therapy as a bridge to allo-HSCT significantly improves long-term leukemia-free survival (LFS) and OS in adult patients with R/R B-ALL, compared with CAR-T monotherapy (20, 21).

Herein, we report a case of an adult patient with early-relapsed, chemorefractory B-ALL who was successfully treated with a dual-targeted CAR-T co-infusion strategy, incorporating a membrane-tethered anti-PD-L1 scFv into the CD22 CAR construct, followed by consolidative allo-HSCT, with comprehensive 7-year follow-up data. This single-case observation provides valuable clinical insights for subsequent preclinical and clinical studies, and demonstrates the potential feasibility and clinical value of this integrated therapeutic regimen for achieving long-term remission in high-risk patients with a historically poor prognosis.

## Case presentation

### Ethics statement

This single-center, retrospective case report was approved by the Ethics Committee of the Second Hospital of Hebei Medical University (approval number: 2017-R207) and conducted in strict adherence with the ethical principles outlined in the Declaration of Helsinki. The CAR-T therapy was administered to the patient under an institutional compassionate use program, as no standard curative treatment options were available for the patient after the development of chemorefractory disease. Written informed consent was obtained from the patient for all treatment procedures, as well as for the publication of this case report and all associated clinical data and imaging. This study is a retrospective observational analysis of a single clinical case, not a prospective interventional clinical trial, and thus was exempt from clinical trial registration requirements in accordance with institutional and national regulatory guidelines for retrospective observational single-case studies.

### Clinical presentation and initial treatment

On May 21, 2018, a 37-year-old woman was admitted to the Department of Hematology, the Second Hospital of Hebei Medical University (Shijiazhuang, China) with a 10-day history of dizziness, fatigue, and fever. Complete blood count (CBC) revealed: white blood cell (WBC) count 82×10^9^/L, hemoglobin (Hb) 54 g/L, platelet (PLT) count 8×10^9^/L.

**Figure 1:**
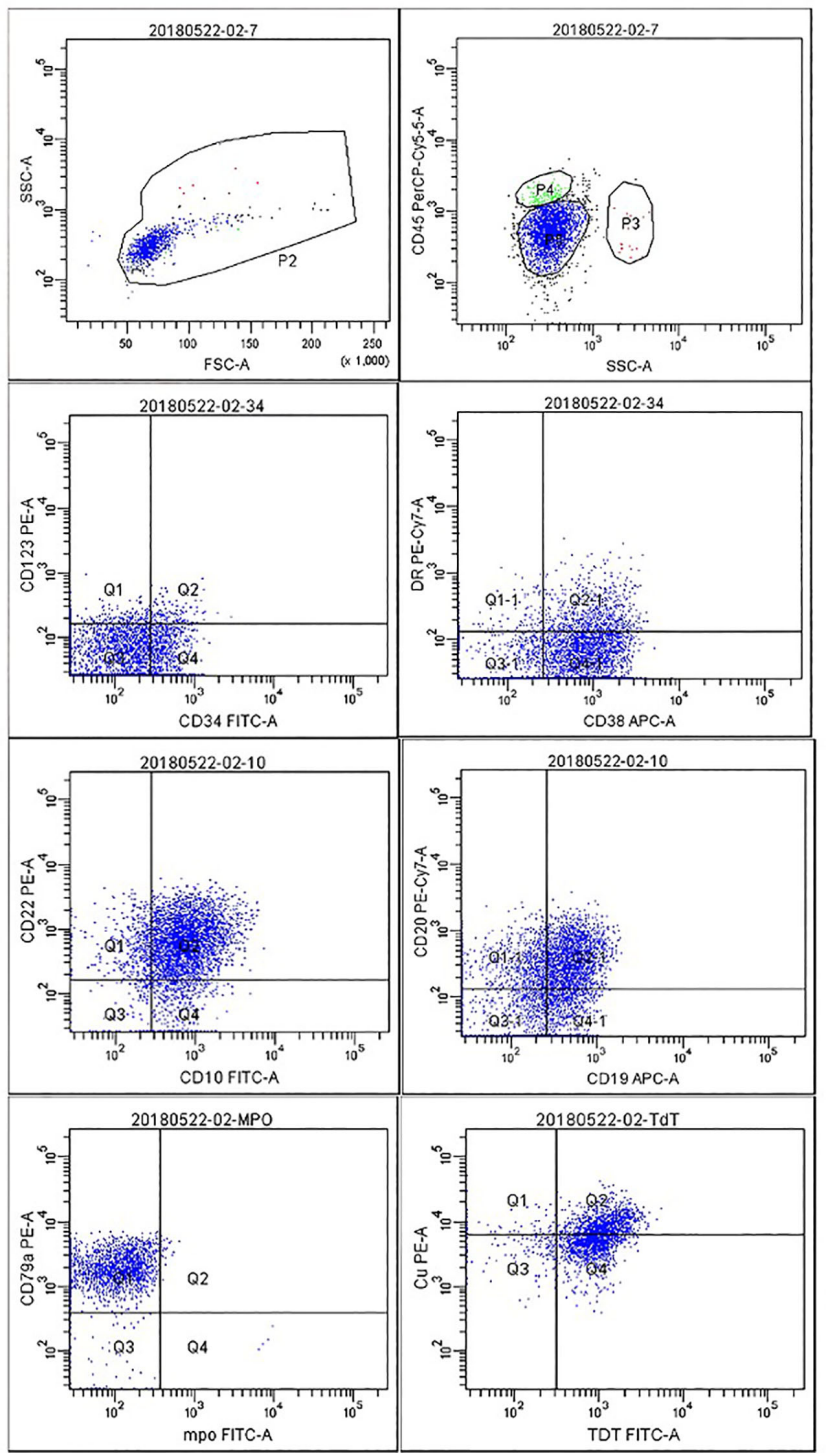
Immunophenotypic analysis confirmed the diagnosis of common B-cell ALL.

Bone marrow (BM) aspirate showed 93% lymphoblasts, with 70% lymphoblasts detected in peripheral blood (PB), suggestive of acute lymphoblastic leukemia (ALL). 8-color multiparameter flow cytometric (MFC) immunophenotyping of the BM aspirate was performed using CD45/side scatter (SSC) gating; 88.6% of nucleated cells fell within the P5 gate, with an immunophenotype positive for CD34 (24.9%), CD38 (86%), CD10 (86.8%), CD22 (89%), CD19 (63.6%), CD20 (65.7%), CD79a (97.9%), and terminal deoxynucleotidyl transferase (TdT, 92.3%), with dim HLA-DR expression and no aberrant expression of other lineage markers, confirming the diagnosis of common B-ALL (**Figure 1**). Quantitative analysis showed a mean fluorescence intensity (MFI) of 15,243 for CD19 and 8,762 for CD22, indicating robust expression of both target antigens.

Comprehensive molecular and cytogenetic diagnostic workup was performed as follows: 8-color MFC with a detection sensitivity of 0.01% was used for immunophenotyping, multiplex leukemia fusion gene screening (including BCR/ABL and Ph-like fusion transcripts) yielded negative results, and conventional G-banding cytogenetic analysis showed a normal female karyotype 46,XX (20). The patient denied any family history of hereditary hematologic diseases, cancer predisposition syndromes, or hematologic malignancies, and no other known high-risk factors for hematologic neoplasms were identified.

The patient was diagnosed with B-ALL and received induction chemotherapy with the VCID regimen (vindesine 3 mg intravenously (IV) on day 1, cyclophosphamide 600 mg IV on days 1–2, idarubicin 20 mg IV on days 1–2, dexamethasone 20 mg IV on days 1–5), and achieved complete remission (CR) after induction. Consolidation chemotherapy was sequentially administered with the VCID regimen (same as induction) and the VCAD regimen (vindesine 3 mg IV on day 1, cyclophosphamide 600 mg IV on days 1–2, cytarabine 200 mg IV on days 1–5, dexamethasone 20 mg IV on days 1–5) plus pegaspargase 3750 IU intramuscularly (IM) on day 6. During consolidation, repeated BM examinations confirmed sustained CR with negative minimal residual disease (MRD) by 8-color MFC. Serial lumbar punctures with intrathecal chemotherapy (dexamethasone 5 mg + methotrexate 10 mg + cytarabine 30 mg) were performed throughout the treatment course; no abnormalities were detected in cerebrospinal fluid (CSF) routine, biochemistry, or flow cytometry tests, and no evidence of central nervous system (CNS) leukemia involvement was observed at any time point.

The patient developed early medullary relapse approximately 5 months after achieving initial CR, confirmed on November 16, 2018. CBC at relapse showed: WBC 2.8 × 10^9^/L, Hb 109 g/L, PLT 115 × 10^9^/L. BM examination revealed 34% leukemic lymphoblasts, with an immunophenotype of CD10^+^CD19^+^CD34^+^CD38^+^CD22^+^CD58^+^CD20^+^CD33^−^, consistent with medullary relapse of the patient’s known B-ALL. The patient received one cycle of salvage chemotherapy with the VCDD regimen (vincristine 2 mg IV on day 1, cyclophosphamide 600 mg IV on days 1–2, daunorubicin 60 mg IV on days 1–3, dexamethasone 20 mg IV on days 1–5). Post-salvage BM re-evaluation showed 16% persistent leukemic lymphoblasts, confirming primary chemorefractory disease. Given the unsatisfactory response to conventional chemotherapy, dual-targeted CAR-T therapy via co-infusion of two independent single-target CAR-T products (CD19 CAR-T and PD-L1-armored CD22 CAR-T) was selected as the salvage treatment. After thorough discussion with the patient and her family, the patient was enrolled in our institutional compassionate use program for this dual-targeted CAR-T therapy.

### CAR-T cell product characteristics and manufacturing

**Figure 2:**
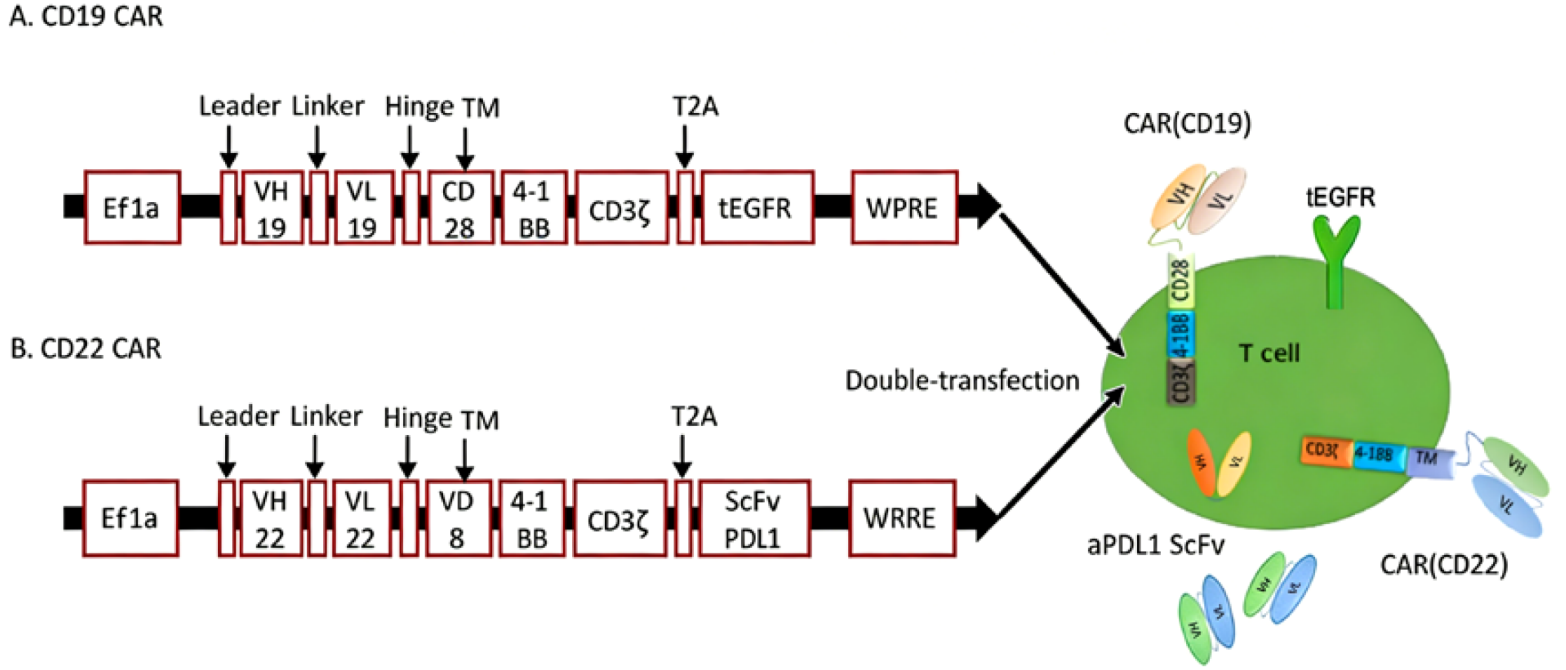
Schematic of CD19/CD22 CAR structures. **(A)** Constructs of CD19 CAR include anti-CD19 scFv, CD28 transmembrane domain, 4-1BB co-stimulation, CD3zeta activation domain, T2A self-cleavage sequence, and truncated EGFR. **(B)** Constructs of CD22 CAR include anti-CD22 scFv, CD8 transmembrane domain, 4-1BB co-stimulation, CD3zeta activation domain, T2A self-cleavage sequence, and anti-PDL1 scFv.

After written informed consent was obtained, 100 mL of PB was collected from the patient, and peripheral blood mononuclear cells (PBMCs) were isolated via leukapheresis for CAR-T cell manufacturing. Hebei Senlang Biotechnology Co., Ltd. (Shijiazhuang, China) was responsible for the design and current Good Manufacturing Practice (cGMP)-compliant production of the CAR-T cell products. The CD19 CAR construct comprised an anti-CD19 single-chain variable fragment (scFv), a CD28 transmembrane domain, a 4-1BB co-stimulatory domain, a CD3ζ signaling domain, a T2A self-cleavage sequence, and a truncated epidermal growth factor receptor (tEGFR) as a cell surface marker (**Figure 2A**). The PD-L1-armored CD22 CAR construct contained an anti-CD22 scFv, a CD8 transmembrane domain, a 4-1BB co-stimulatory domain, a CD3ζ signaling domain, a T2A self-cleavage sequence, and a membrane-tethered anti-PD-L1 scFv for localized immune checkpoint modulation (**Figure 2B**).

T cells were isolated from the leukapheresis product using magnetic bead-based selection and activated with anti-CD3/CD28 antibodies. After 48 hours of activation, T cells were transduced with lentiviral vectors at a multiplicity of infection (MOI) of 5. The lentiviral packaging system included four lentiviral plasmids (pLenti-EF1-CAR19, pMD2.G, pLenti-EF1-CAR22, and psPAX2), which were co-transfected into 293FT cells using JetPRIME reagent. Vector copy number analysis confirmed 2.3 ± 0.4 vector copies per transduced cell. High transduction efficiency was achieved for both products (CD19 CAR-T: 96%; CD22 CAR-T: 71.5%).

The final products underwent rigorous quality control, including sterility testing, endotoxin quantification (<0.25 EU/mL), and functional validation via *in vitro* cytotoxicity assays against CD19^+^CD22^+^ NALM-6 cells. The final products exhibited >95% cell viability and >85% specific lysis of target cells at an effector:target (E:T) ratio of 5:1. This co-infusion strategy was selected based on preclinical validation demonstrating superior anti-leukemic efficacy compared with sequential infusion of single-target CAR-T products, while circumventing the ~25% co-transduction efficiency limit associated with dual CAR expression in a single T cell, and enabling independent optimization of each CAR product and flexible dose adjustment (12, 13).

The CAR-T cell products were manufactured in compliance with cGMP standards by Hebei Senlang Biotechnology Co., Ltd. (Shijiazhuang, China). The full proprietary manufacturing workflow is the commercial confidential information of the manufacturer; however, all key parameters relevant to clinical application—including CAR construct design, vector copy number, transduction efficiency, product quality control criteria, and infusion doses—are fully provided herein to support the reproducibility of the clinical regimen. Of note, this study focuses on the clinical efficacy and safety profile of the regimen, not the development of the CAR-T cell manufacturing platform.

### Clinical course following CAR-T infusion

The patient received lymphodepleting chemotherapy with cyclophosphamide (30 mg/kg/day IV on days −3 to −2) and fludarabine (30 mg/m²/day IV on days −4 to −2). On December 7, 2018 (day 0), the patient received co-infusion of 5.0×10^5^ CD19 CAR-T cells/kg and 3.1×10^5^ PD-L1-armored CD22 CAR-T cells/kg over 30 minutes, with no immediate adverse reactions.

**Figure 3:**
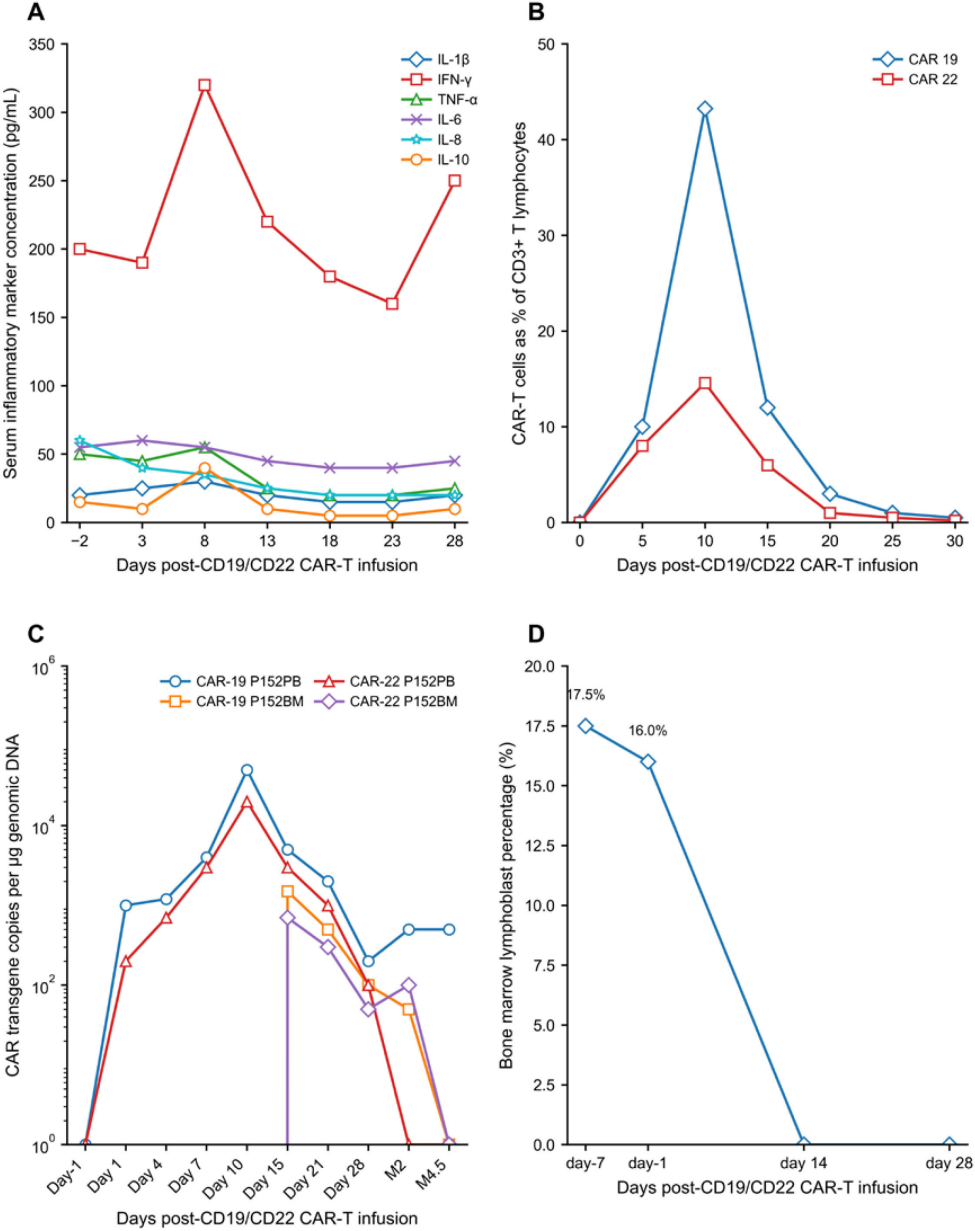
Changes following CAR-T cell infusion: **(A)** concentrations of cytokines in serum. **(B)** Expression of CD19/22 CAR-T cells detectable by flow cytometry in peripheral blood. Day 0 is the day of CD19/CD22 CAR-T cell infusion. **(C)** The presence of CAR-T 19/22 cells in peripheral blood and bone marrow as assessed by qPCR. **(D)** Number of lymphoblasts in the bone marrow.

After CAR-T cell infusion, the patient developed grade 1 cytokine release syndrome (CRS) in accordance with the ASTCT consensus criteria, characterized by low-grade fever (38.3 °C) on day 8 post-infusion, which resolved within 24 hours with supportive care including non-steroidal anti-inflammatory drugs. No immune effector cell-associated neurotoxicity syndrome (ICANS), per ASTCT criteria, was observed. Serum cytokine profiling showed transient elevation of inflammatory biomarkers, including interleukin-6 (IL-6, peak 128.6 pg/mL), interferon-γ (IFN-γ), and C-reactive protein (CRP, peak 156.7 mg/L), with all peaks occurring between days 8 and 10 (**Figure 3A**). This mild CRS profile may be attributed to the relatively low disease burden at treatment initiation and the immunomodulatory effects of the membrane-tethered anti-PD-L1 scFv in the CD22 CAR construct, which may have mitigated excessive inflammatory responses via spatially restricted PD-L1 blockade without systemic immune overactivation (22).

CAR-T cell expansion was dynamically monitored by flow cytometry and quantitative polymerase chain reaction (qPCR). Circulating CAR-T cells reached their peak expansion on day 10 post-infusion, with CD19 CAR-T cells accounting for 43.25% and CD22 CAR-T cells accounting for 14.58% of circulating CD3^+^ T lymphocytes (**Figure 3B**), consistent with the data presented in **Table 1**. The differential expansion kinetics of the two CAR-T products may be explained by their distinct molecular designs: the CD19 CAR incorporates a CD28 transmembrane domain that drives rapid early T cell expansion, while the CD22 CAR contains a CD8 transmembrane domain and a 4-1BB co-stimulatory domain that favors long-term T cell persistence, resulting in complementary anti-leukemic kinetics (23). qPCR confirmed robust *in vivo* expansion of both CAR-T products, with 2.78×10^4^ copies/μg DNA for the CD19 CAR and 1.24×10^4^ copies/μg DNA for the CD22 CAR detected in peripheral blood on day 10 post-infusion (**Figure 3C**).

**Table 1:** Hematologic and CAR-T cell monitoring parameters during treatment course.

Parameter	Pre-car-t (day -1)	Day 10 (peak car-t)	Day 14 (remission)	Day +30 (post-hsct)
WBC (×10^9^/L)	2.8	1.2	6.7	3.9
Hb (g/L)	109	83	101	92
PLT (×10^9^/L)	115	38	109	85
BM Blasts (%)	16	8.5	1	0
CD19 CAR-T (%)	–	43.25	35.1	8.75
CD22 CAR-T (%)	–	14.58	9.32	ND
IL-6 (pg/mL)	5.3	128.6	22.4	7.8
CRP (mg/L)	8.2	156.7	34.5	6.3

By day 14 post-infusion, complete hematologic recovery was observed, with WBC 6.7×10^9^/L, Hb 101 g/L, and PLT 109×10^9^/L. BM examination showed only 1% residual lymphoblasts, with no detectable CD19^+^CD22^+^ leukemic blasts by 8-color MFC, confirming MRD-negative CR (**Figure 3D**). The 8-color flow cytometric MRD panel included CD19, CD22, CD10, CD34, CD38, CD58, CD20, and CD45, with a detection sensitivity of 0.01%. NGS of IGH gene rearrangements further confirmed MRD negativity, with a LOD of 10^−6^. The rapid clearance of leukemic blasts achieved with relatively low CAR-T cell doses in this case demonstrates that the combination of dual-targeted antigen recognition and intrinsic PD-L1 checkpoint modulation endows CAR-T cells with synergistic and potent anti-leukemic activity (24).

### Allogeneic hematopoietic stem cell transplantation

Given the extremely high risk of disease relapse in patients with early-relapsed, chemorefractory B-ALL even after achieving MRD-negative CR, HLA-haploidentical allo-HSCT was planned as consolidative curative therapy. The patient had an HLA 6/10-matched sibling donor (younger brother, blood type O RhD+; recipient blood type B RhD+), who provided written informed consent for peripheral blood stem cell (PBSC) donation. A fully HLA-matched related or unrelated donor was not available, and the haploidentical donor was selected due to the urgent clinical need for consolidative transplantation after CAR-T-induced remission.

The patient underwent HLA-haploidentical sibling PBSC transplantation combined with adjuvant infusion of third-party umbilical cord blood (UCB) mononuclear cells (total nucleated cells [TNC] 1.0×10^7^/kg). The UCB unit was obtained from the Shandong Cord Blood Hematopoietic Stem Cell Bank, which is fully compliant with national regulatory standards for clinical UCB use. Adjuvant UCB mononuclear cell infusion has been shown to accelerate post-transplant immune reconstitution, regulate the post-transplant immune microenvironment, enhance the GVL effect, and potentially reduce the risk of transplant-related complications (25).

The patient underwent formal disease re-evaluation on December 21, 2018 (14 days post-CAR-T infusion), which confirmed MRD-negative CR in both BM and PB; sustained MRD negativity was reconfirmed in the final pre-transplant assessment. Approximately 2.5 months after CAR-T infusion (February 27–28, 2019), the patient received the allogeneic graft infusion, which contained 10.44×10^8^/kg total mononuclear cells (CD34^+^ cell dose: 7.61×10^6^/kg) from the HLA-haploidentical sibling donor, combined with 1.2×10^7^/kg nucleated cells from the third-party UCB unit.

Myeloablative conditioning was initiated on day −10 pre-transplantation, with the following regimen: cytarabine (4 g/m²/day IV on days −10 to −9), busulfan (3.2 mg/kg/day IV on days −8 to −6), cyclophosphamide (1.8 g/m²/day IV on days −5 to −4), and rabbit anti-thymocyte globulin (2.5 mg/kg/day IV on days −4 to −2). Graft-versus-host disease (GVHD) prophylaxis consisted of cyclosporine A (target trough level: 150–200 ng/mL), mycophenolate mofetil (15 mg/kg orally twice daily), and short-course methotrexate (15 mg/m² IV on day +1, 10 mg/m² IV on days +3, +6, and +11).

Neutrophil engraftment (absolute neutrophil count [ANC] >0.5×10^9^/L) occurred on day +12 post-transplantation, and platelet engraftment (platelet count >20×10^9^/L) occurred on day +14, consistent with the median engraftment timeline for haploidentical HSCT at our institution. BM examination on day +30 confirmed complete morphologic remission, with no detectable leukemic blasts and negative MRD by MFC. Chimerism analysis by STR on day +30 demonstrated complete donor-type hematopoiesis (99.86% donor chimerism). Quantitative monitoring of CAR-T cells showed that CD19 CAR-T cells persisted for approximately 4.5 months post-infusion, while PD-L1-armored CD22 CAR-T cells became undetectable at 2 months post-infusion. The patient was discharged with stable hematopoiesis and followed up regularly in our outpatient clinic.

### 7-year follow-up

Regular follow-up assessments were performed at 1, 3, 6, 12, 24, 36, 48, 60, 72, and 84 months post-transplantation. At each visit, comprehensive evaluations were performed, including CBC, donor chimerism analysis, MRD testing by MFC, complete metabolic panel, and detailed physical examination. BM aspirations with cytogenetic analysis and NGS-based IGH rearrangement MRD assessment were performed at 3, 6, 12, 24, 36, 48, 60, and 72 months post-transplantation to screen for disease recurrence.

At 84 months (7 years) post-transplantation, the patient maintained 100% complete donor chimerism, sustained MRD-negative remission, and normal hematologic parameters (**Table 2**). Comprehensive physical examination showed no evidence of extramedullary disease. No acute or chronic GVHD was observed during the entire 7-year follow-up period. The patient did not require any immunosuppressive therapy beyond the planned tapering of cyclosporine A, which was completely discontinued by day +180 post-transplantation. No secondary malignancies or significant late treatment-related adverse events associated with prior CAR-T therapy or allo-HSCT were detected during the 7-year follow-up. Complete immune reconstitution was achieved by 24 months post-transplantation, with normalization of CD4^+^ T cell counts and serum immunoglobulin levels.

**Table 2:** Long-term follow-up parameters from 1 to 7 years post-transplantation.

Parameter	1 year	2 years	3 years	4 years	5 years	6 years	7 years
WBC (×10^9^/L)	5.1	5.8	6.3	6.5	6.6	6.7	6.8
Hb (g/L)	118	125	132	135	136	137	139
PLT (×10^9^/L)	145	168	185	192	198	202	205
Donor Chimerism (%)	100	100	100	100	100	100	100
CD4^+^ T cells (cells/μL)	310	485	590	620	650	680	710
CD19^+^ B cells (cells/μL)	85	195	310	345	375	410	430
Serum IgG (g/L)	6.8	8.1	9.5	10.2	10.8	11.3	11.7
ECOG Performance Status	0						
GVHD Grade	0						
MRD Status	Negative						

The patient maintained excellent functional status throughout the follow-up period, returning to full-time employment at 12 months post-transplantation. Quality of life assessment using the FACT-Leu questionnaire showed progressive improvement, reaching near-normal values by 24 months and remaining stable thereafter (total score 158/172 at the 7-year follow-up). Comprehensive cardiovascular assessment, including echocardiography, showed preserved left ventricular ejection fraction (62%) with no evidence of heart failure. Neurocognitive testing demonstrated normal cognitive function across all assessed domains. Endocrine evaluation showed normal thyroid, adrenal, and gonadal function. This 7-year comprehensive follow-up confirms the durability of remission and favorable long-term safety profile of this integrated therapeutic strategy (26).

## Discussion

Durable remission exceeding 7 years remains exceptionally rare in adult patients with early-relapsed, chemorefractory B-ALL, particularly in those undergoing haploidentical allo-HSCT. Pivotal clinical trials of CD19-targeted CAR-T monotherapy have reported a 5-year event-free survival (EFS) rate of only 20–30% in adult patients with R/R B-ALL (5, 6), while studies of dual-targeted CD19/CD22 CAR-T therapy have reported 3-year EFS rates of 40–50% in similar high-risk populations (24). For patients with early relapse within 6 months of first complete remission (CR1), the 5-year overall survival (OS) rate is generally less than 10% with conventional salvage chemotherapy (1). To contextualize the long-term outcomes of this case, we systematically compared our findings with previously published clinical studies of CAR-T therapy with or without subsequent allo-HSCT in adult patients with R/R B-ALL (**Supplementary Table 1**). Briefly, most published prospective trials and case series of CD19/CD22 dual-targeted CAR-T therapy in adult high-risk B-ALL have reported a median follow-up of 12 to 36 months, with 3-year OS rates ranging from 40% to 60% (24, 27, 28). For adult patients with early-relapsed, chemorefractory B-ALL who received CAR-T therapy bridging to haploidentical allo-HSCT, the majority of published studies have a maximum follow-up of less than 5 years, with 5-year OS rates below 30% (1, 29). While several case reports have described long-term remission exceeding 5 years after CAR-T therapy for B-ALL (30, 31), reports of 7-year sustained MRD-negative EFS in adult patients with early-relapsed, chemorefractory B-ALL treated with PD-L1-armored dual-targeted CAR-T bridging to haploidentical allo-HSCT remain extremely limited in the existing literature. The 7-year MRD-negative EFS observed in this case thus provides a valuable reference for the optimization of integrated cellular therapy strategies for this historically poor-prognosis patient population.

In this case, we describe an integrated therapeutic strategy consisting of CD19/PD-L1-armored CD22 dual-targeted CAR-T cell co-infusion, followed by HLA-haploidentical allo-HSCT with adjuvant third-party UCB mononuclear cell infusion, which resulted in 7-year EFS in an adult patient with early-relapsed, chemorefractory B-ALL, providing a valuable clinical reference for the management of this high-risk population. For patients with early relapse within 6 months after CR1, the long-term survival rate is generally less than 10% even after intensive salvage therapy, with an extremely poor overall prognosis (32). The integrated strategy applied in this study specifically addresses three key barriers to long-term remission in high-risk B-ALL: the risk of antigen escape, the immunosuppressive TME, and insufficient consolidation before transplantation.

The co-infusion strategy of separately manufactured CD19 and CD22 CAR-T products offers distinct therapeutic advantages over alternative dual-targeted approaches (such as tandem CAR or bicistronic CAR constructs in a single T cell). The differential expansion kinetics observed in this case (CD19 CAR-T accounting for 43.25% and CD22 CAR-T for 14.58% of circulating T cells at day 10 post-infusion) reflects the strategic molecular designs of the two constructs: the CD19 CAR incorporates a CD28 transmembrane domain that drives rapid early T cell expansion, while the CD22 CAR contains a CD8 transmembrane domain and a 4-1BB co-stimulatory domain that favors long-term T cell persistence, resulting in complementary anti-leukemic kinetics. This co-infusion approach circumvents the technical limitations of co-transduction strategies, where vector capacity and transduction efficiency constrain stable dual CAR expression in a single T cell (33). The rapid achievement of MRD-negative CR by day 14 post-infusion demonstrates the clinical efficacy of simultaneously targeting two lineage-restricted B-cell antigens to overcome clonal heterogeneity, which is particularly critical in early-relapsed disease where antigen escape is a major driver of therapeutic resistance. This dual-targeted strategy has demonstrated remarkable efficacy in pediatric populations, with a 100% MRD-negative CR rate reported in children with B-ALL who relapsed after prior CD19 CAR-T therapy (23).

The integration of a membrane-tethered anti-PD-L1 scFv directly into the CD22 CAR construct represents a precision strategy for spatially restricted immune checkpoint modulation within the TME. Unlike systemic immune checkpoint blockade, which carries the risk of uncontrolled systemic inflammation and off-target autoimmunity, the membrane-bound anti-PD-L1 scFv used in this regimen delivers localized immune modulation exclusively within the TME where CAR-T cells engage with target leukemic blasts (22).

Mechanistically, preclinical and clinical studies have demonstrated that the PD-L1 scFv expressed on the CAR-T cell surface competitively binds to PD-L1 in the TME and blocks the inhibitory PD-1/PD-L1 interaction, with three proposed core biological effects: (1) reversal of the terminal differentiation and functional exhaustion of tumor-reactive T cells via restoration of their proliferative capacity, cytokine secretion, and cytotoxic activity (34); (2) remodeling of the immunosuppressive TME via inhibition of the recruitment and suppressive function of regulatory T cells (Tregs) and tumor-associated macrophages, thereby enhancing effector T cell infiltration into tumor tissues (15, 35); (3) promotion of long-term tumor-specific immune memory via preservation of memory T cell stemness (34). These effects collectively reprogram the immunosuppressive TME into an immune-permissive state (16, 36, 37).

In this case, we observed robust *in vivo* expansion of both CAR-T products, rapid achievement of MRD-negative CR, mild grade 1 CRS with no ICANS, successful hematopoietic engraftment after haploidentical allo-HSCT, and long-term sustained remission with no GVHD. These clinical findings are consistent with the above-described mechanistic rationale, and support the hypothesis that the PD-L1-armored CAR-T construct not only exerts direct anti-leukemic effects, but also remodels the host immune state, clears minimal residual disease, and optimizes the pre-transplant immune microenvironment, thereby creating favorable conditions for successful allo-HSCT engraftment and long-term disease control. This forms a sequential mechanistic framework of CAR-T-mediated immune reprogramming followed by improved transplant conditions and enhanced long-term outcomes.

Notably, several critical limitations of this mechanistic interpretation must be explicitly acknowledged. First, this study is a single-center retrospective observational analysis of a single patient without a parallel control group, which is an inherent limitation of the case report study design. Thus, the causal relationship between the PD-L1 armored module and the observed clinical outcomes cannot be definitively established, and the findings are strictly hypothesis-generating rather than conclusive. Second, this interpretation is based on preclinical evidence from existing literature and the clinical observations in this case, with no direct *in vitro* or *in vivo* experimental validation performed in this study. Third, the specific independent contribution of the anti-PD-L1 scFv component to the observed clinical outcomes cannot be isolated from the effects of dual-targeted antigen recognition and subsequent allo-HSCT in this single case. The specific role of the PD-L1 armored module in this regimen requires further validation in prospective, multi-center clinical trials with larger patient cohorts.

Accumulating clinical evidence has confirmed the feasibility and efficacy of PD-L1-modified CAR-T therapy combined with allo-HSCT in the treatment of high-risk hematologic malignancies (15, 17). Consistent with our observations, preclinical studies have demonstrated that localized PD-L1 blockade via membrane-tethered scFv can enhance CAR-T cell effector function and persistence in the immunosuppressive TME, without the systemic toxicities associated with antibody-based immune checkpoint inhibitors (22). Clinical studies have also shown that PD-1/PD-L1 pathway blockade can reverse T cell exhaustion, remodel the tumor immune microenvironment, and improve the efficacy of CAR-T therapy in R/R B-cell malignancies (15). Taken together, these published data support the biological plausibility of our mechanistic hypothesis, and our clinical observations provide preliminary in-human evidence for the potential value of this integrated regimen in high-risk B-ALL patients.

This precision localized PD-L1 blockade approach likely contributed to the mild grade 1 CRS profile and rapid inflammatory resolution observed in this case, which contrasts with the higher incidence of severe CRS reported in conventional dual-targeted CAR-T approaches (38). Notably, no acute or chronic GVHD was observed during the entire 7-year follow-up period despite the significant HLA disparity (6/10 match) between the donor and recipient. This finding further supports the hypothesis that localized PD-L1 blockade may modulate alloreactive immune responses while preserving the beneficial GVL effect, which is consistent with previous reports suggesting that CAR-T therapy is associated with a lower risk of GVHD compared with conventional donor lymphocyte infusion (DLI) (39).

The 2.5-month interval between CAR-T infusion and haploidentical allo-HSCT represents a strategically optimized therapeutic window informed by contemporary clinical evidence. While adverse factors such as TP53 mutation and high pre-infusion tumor burden have been reported to negatively impact the response rate to CAR-T therapy (17), the rapid achievement of MRD-negative CR in our patient provided an ideal opportunity for consolidative allo-HSCT before the emergence of potential antigen escape or resistant clones. Notably, the absence of GVHD despite significant HLA disparity suggests that prior CAR-T therapy may have favorably modulated the alloreactive immune landscape through lymphodepletive effects or regulatory T cell modulation. This observation aligns with previous evidence demonstrating that donor-derived CAR-T therapy can improve survival outcomes in patients with B-ALL relapsed after allo-HSCT (27).

The 7-year comprehensive follow-up in this case provides valuable insights into the long-term outcomes of this integrated cellular therapy strategy. Our patient achieved complete immune reconstitution by 24 months post-transplantation, with normalization of CD4^+^ T-cell counts (710 cells/μL), complete recovery of serum immunoglobulin levels (IgG 11.7 g/L), and full restoration of CD19^+^ B-cell counts (430 cells/μL). This immune reconstitution profile contrasts with the prolonged B-cell aplasia typically observed after CD19-directed CAR-T monotherapy (40). The accelerated functional immune recovery likely contributed to the patient’s excellent quality of life (FACT-Leu total score 158/172 at 7 years) and the absence of severe opportunistic infections throughout the follow-up period. In addition, no cardiovascular, neurocognitive, or endocrine sequelae were detected during the 7-year follow-up, demonstrating the favorable long-term safety profile of this integrated strategy compared with conventional intensive chemotherapy or radiation-based regimens.

Notably, we observed that CD19 CAR-T cells were detectable in peripheral blood for approximately 4.5 months post-infusion, while PD-L1-armored CD22 CAR-T cells became undetectable at 2 months post-infusion. This difference in *in vivo* persistence between the two CAR-T products may be closely related to the structural and functional differences between the two CAR constructs, as well as the biological characteristics of the target antigens.

The PD-L1-armored CD22 CAR-T cells were engineered to additionally express a membrane-tethered PD-L1 scFv, which is theoretically designed to enhance anti-tumor activity and T cell persistence by blocking the PD-1/PD-L1 inhibitory axis and reversing T cell exhaustion (16, 22). However, this exogenous functional module may introduce unintended negative effects that limit the long-term persistence of CD22 CAR-T cells:

First, the additional expression of the PD-L1 scFv may interfere with the normal protein folding, membrane surface expression, and proximal signal transduction of the CD22 CAR molecule, thereby impairing the initial expansion capacity and long-term survival potential of CD22 CAR-T cells (33). Previous structural studies have demonstrated that the addition of exogenous functional modules to CAR constructs can alter the spatial conformation of the CAR molecule, reduce its binding affinity to the target antigen, and impair downstream signaling activation, ultimately limiting T cell expansion and persistence (33).

Second, the exogenous PD-L1 scFv, as a non-native protein fragment, may be recognized as a foreign antigen by the host immune system, triggering an anti-CAR immune response and accelerating the *in vivo* clearance of PD-L1-armored CD22 CAR-T cells. This is a well-documented mechanism limiting the persistence of CAR-T cells expressing non-native protein domains (38).

Third, although PD-L1 blockade relieves the inhibitory signal in the TME, it may also over-activate T cell receptor signaling pathways, disrupt the metabolic homeostasis and activation threshold of T cells, and lead to earlier terminal differentiation and functional exhaustion of CD22 CAR-T cells compared with CD19 CAR-T cells (34). Preclinical studies have shown that excessive T cell activation can drive terminal differentiation of effector T cells, reduce the proportion of memory T cell subsets, and ultimately limit the long-term persistence of CAR-T cells *in vivo* (34).

In contrast, the classic CD19 CAR-T construct without an exogenous armored module has a simpler and more stable structure, which may reduce the risk of immune recognition and structural interference. Meanwhile, CD19 antigen has a wider and more stable expression profile on B-ALL cells compared with CD22, which can provide more sustained and stable antigen stimulation to maintain the proliferative activity and functional stability of CD19 CAR-T cells (7, 9). These factors collectively contributed to the superior long-term persistence of CD19 CAR-T cells observed in this case. This observation provides important clinical clues for the structural optimization of armored CAR-T constructs and the improvement of long-term persistence of dual-targeted CAR-T therapy in future preclinical and clinical studies.

Several limitations of this study warrant explicit acknowledgment. First and foremost, this is a single-center, retrospective case report of a single patient, and the findings are strictly hypothesis-generating rather than conclusive. The clinical outcomes observed in this individual patient cannot be generalized to the broader patient population. Second, the lack of a parallel control group is an inherent limitation of the case report study design. Third, the mechanistic interpretation of the PD-L1-armored module’s role is based on preclinical literature and our clinical observations, with no direct *in vitro* or *in vivo* experimental validation performed in this study; thus, the precise independent contribution of the anti-PD-L1 scFv component to the observed clinical outcomes cannot be isolated and remains to be elucidated. Fourth, as a single-case observation, this study cannot definitively define the patient population most likely to benefit from this integrated therapeutic strategy. Future large-scale, multi-center prospective clinical trials are required to identify the clinical and biological characteristics of patients who may derive the greatest benefit from this regimen, as well as to optimize the dosing ratio of CD19 to PD-L1-armored CD22 CAR-T cells and the optimal interval between CAR-T infusion and consolidative allo-HSCT (41, 42). Nevertheless, accumulating evidence supports the efficacy of multi-targeted CAR-T approaches in preventing antigen escape, with dual-targeted CD19/CD22 CAR-T therapy showing particular promise in high-risk B-ALL populations (12, 43, 44). The 7-year sustained MRD-negative remission without chronic GVHD or significant late toxicities observed in this case is notable in the context of the historically poor prognosis of early-relapsed chemorefractory B-ALL, and suggests that this specific combination of dual-targeted CAR-T co-infusion with integrated PD-L1 checkpoint modulation followed by allo-HSCT warrants further investigation in high-risk B-ALL.

Recent evidence has clarified the patient population most likely to benefit from consolidative allo-HSCT after CAR-T therapy. Patients with complex karyotypes, adverse genetic markers, and high pre-infusion MRD levels are at high risk of relapse after CAR-T monotherapy (42). Our patient’s early relapse with chemorefractory disease firmly placed her in this high-risk category, justifying the use of this integrated therapeutic approach. The “sandwich strategy” of sequential CD19/CD22 CAR-T therapy combined with autologous HSCT, as pioneered in Philadelphia chromosome-negative B-ALL, has demonstrated an impressive 2-year overall survival rate of 97% (21). Our approach extends this concept by incorporating allogeneic rather than autologous HSCT to achieve a more potent GVL effect, and integrating immune checkpoint modulation directly within the CAR construct to enhance the anti-leukemic efficacy of CAR-T cells.

The favorable outcomes observed in this case also align with emerging data on the role of CAR-T cell functional quality in determining long-term efficacy. Fabrizio et al. demonstrated an excellent 24-month overall survival rate of 80% in pediatric and young adult patients with relapsed/refractory B-ALL who received CAR-T therapy followed by T cell-depleted allo-HSCT (28). Similarly, Liu et al. found that patients who received CD19 CAR-T therapy bridging to allo-HSCT had significantly improved 1-year overall survival (70%) and 1-year leukemia-free survival (95%) compared with those who received CAR-T monotherapy (45).

From a mechanistic perspective, the sustained long-term remission in this patient is driven by the successful establishment of durable GVL effects. Although the infused CAR-T cells were gradually cleared *in vivo*—with CD19 CAR-T cells becoming undetectable at 4.5 months post-infusion and PD-L1-armored CD22 CAR-T cells undetectable at 2 months—their core role in inducing deep MRD-negative complete remission established a critical immunological foundation for the subsequent allo-HSCT (46). This clinical observation aligns with recent clinical evidence confirming that consolidative allo-HSCT after CAR-T-induced remission can achieve sustained long-term disease control in patients with high-risk B-ALL (47).

The integration of PD-L1 checkpoint blockade represents a particularly innovative aspect of our approach. Preclinical and clinical evidence has established that Tregs in the TME play a significant role in limiting the efficacy of CAR-T therapy, with higher Treg frequencies correlating with shorter relapse-free survival and overall survival (35). The anti-PD-L1 scFv incorporated into our CD22 CAR construct likely mitigated this immunosuppressive influence of Tregs, contributing to the observed mild CRS profile and robust *in vivo* expansion kinetics of CAR-T cells. This strategy builds upon the established principles of immune checkpoint inhibition while avoiding the systemic toxicities associated with conventional antibody-based immune checkpoint inhibitors (48).

For patients with extramedullary disease, a particularly challenging clinical scenario in B-ALL, our approach may offer distinct advantages. Li et al. reported a case of extramedullary relapsed B-ALL successfully treated with CAR-T therapy bridging to unrelated cord blood transplantation, but the patient ultimately relapsed after 6 months (49). While the initial response was robust in that case, the addition of integrated PD-L1 checkpoint modulation in our approach may further enhance the durability of remission in such high-risk clinical presentations.

The 7-year EFS reported in this case is significantly longer than the outcomes described in most published series of high-risk B-ALL. Yang et al. demonstrated comparable transplant outcomes in patients receiving haploidentical HSCT after CAR-T-induced CR versus chemotherapy-induced CR, but with a median follow-up of only 31.0 months (29). Similarly, Truong et al. highlighted the evolving role of allo-HSCT in pediatric ALL in the era of immunotherapy, noting that novel immunotherapeutic agents can achieve MRD-negative remission with reduced toxicity, but long-term follow-up data remained limited (31).

The exceptional durability of remission observed in our case may be attributed to the synergistic effects of multiple therapeutic components: (1) dual-targeted CAR-T cells targeting CD19 and CD22 to reduce the risk of antigen escape; (2) integrated PD-L1 checkpoint modulation to enhance CAR-T effector function and reprogram the immunosuppressive TME; (3) appropriately timed HLA-haploidentical allo-HSCT with adjuvant UCB mononuclear cell infusion to provide additional GVL effects, accelerate immune reconstitution, and reduce the risk of transplant-related complications. This multimodal strategy addresses the multiple resistance mechanisms that typically limit the efficacy of single-modality approaches in high-risk B-ALL.

From a practical standpoint, the relatively low CAR-T cell doses employed in this case (5.0 × 10^5^ CD19 CAR-T cells/kg and 3.1 × 10^5^ PD-L1-armored CD22 CAR-T cells/kg) achieved deep MRD-negative CR with minimal toxicity, suggesting that rational CAR design may be more critical than high cell doses for therapeutic efficacy. This observation aligns with findings from the FasT CAR-T platform, which demonstrated that CD19 F-CAR-T cells manufactured within a single day achieved superior remission rates compared with conventional manufacturing approaches (50).

In summary, this case report details an adult patient with early-relapsed, chemorefractory B-ALL who achieved 7-year MRD-negative sustained remission after treatment with co-infusion of CD19 CAR-T and PD-L1-armored CD22 CAR-T cells, followed by consolidative HLA-haploidentical allo-HSCT with adjuvant third-party UCB mononuclear cell infusion. The 7-year EFS, complete immune and functional recovery, and favorable long-term safety profile observed in this case provide valuable clinical insights for the management of this historically poor-prognosis patient population. These findings provide a preliminary rationale for future prospective clinical trials evaluating this multimodal therapeutic strategy in patients with high-risk R/R B-ALL, with careful attention to optimal patient selection, CAR-T dosing, and timing of consolidative allo-HSCT. The therapeutic strategy described herein offers a potential treatment option for patients with chemorefractory B-ALL who have no remaining standard curative treatment options, and establishes a preliminary framework for integrating armored CAR-T cellular immunotherapies with consolidative allo-HSCT. Future preclinical and clinical research should focus on identifying biomarkers predictive of long-term treatment response, optimizing the structure of armored CAR-T constructs to improve the long-term persistence of dual-targeted CAR-T cells, and defining the optimal sequence of cellular therapy and transplantation to maximize cure rates while minimizing treatment-related morbidity.

## Data Availability

The original contributions presented in the study are included in the article/**Supplementary Material**. Further inquiries can be directed to the corresponding author.

## Supplementary material

Supplementary Table 1 Summary of clinical and preclinical studies of CAR-T therapy with or without allogeneic hematopoietic stem cell transplantation for relapsed/refractory b-cell malignancies.
