# Supplementary material for: PD-L1-armored CD19/CD22 dual-targeted CAR-T cell co-infusion bridging to allogeneic hematopoietic stem cell transplantation achieves 7-year sustained remission in an adult patient with early relapsed, chemorefractory B-cell acute lymphoblastic leukemia: a case report
Source: Front Immunol. 2026 Apr 27;17:1797562. doi: 10.3389/fimmu.2026.1797562 (PMC13158182; doi:10.3389/fimmu.2026.1797562)
Supplement: Supplementary Table 1 — Summary of clinical and preclinical studies of CAR-T therapy with or without allogeneic hematopoietic stem cell transplantation for relapsed/refractory b-cell malignancies. [file Table1.docx]

**Supplementary Table S1**

**Supplementary Table S1.** Summary of Clinical and Preclinical Studies of CAR-T Therapy with or without Allogeneic Hematopoietic Stem Cell Transplantation for Relapsed/Refractory B-Cell Malignancies

| Study ID | First Author | Year | Study Type | Sample Size | CAR-T Construct & Modification | Bridging allo-HSCT | Median Follow-up | Key Efficacy Outcomes | Key Safety Outcomes | Reference No. |
| --- | --- | --- | --- | --- | --- | --- | --- | --- | --- | --- |
| 1 | Maude | 2014 | Prospective clinical trial | 30 | CD19-targeted CAR-T with 4-1BB costimulatory domain | Not specified | 6.1 months | 90% of patients achieved complete remission (CR); sustained remissions observed in 67% of responders | Grade 3-4 cytokine release syndrome (CRS) in 27% of patients; grade 3-4 neurotoxicity in 13% of patients | 1 |
| 2 | Rafiq | 2018 | Preclinical in vivo study + translational research | N/A (murine model) | CAR-T engineered to secrete PD-1-blocking scFv; dual targeting of tumor antigen and PD-1/PD-L1 pathway | Not applicable | N/A | Enhanced anti-tumor efficacy and prolonged survival in murine models; reduced T cell exhaustion in tumor microenvironment | No observed systemic immune-related adverse events in murine models | 2 |
| 3 | Park | 2018 | Prospective clinical trial | 53 | CD19-targeted CAR-T with 4-1BB costimulatory domain | 47% of patients underwent allo-HSCT after CAR-T | 29 months | 83% of patients achieved CR; 5-year overall survival (OS) rate of 59%; 5-year event-free survival (EFS) rate of 44% | Grade 3-4 CRS in 26% of patients; grade 3-4 neurotoxicity in 15% of patients | 3 |
| 4 | Fry | 2018 | Prospective phase 1/2 clinical trial | 21 | CD22-targeted CAR-T with 4-1BB costimulatory domain | 38% of patients underwent allo-HSCT after CAR-T | 13.4 months | 73% of patients achieved CR; 61% of patients with prior CD19 CAR-T failure achieved CR | Grade 3-4 CRS in 24% of patients; grade 3 neurotoxicity in 5% of patients | 4 |
| 5 | Wang | 2019 | Narrative review | N/A | Summary of CTLA-4/PD-L1/PD-1-based immune checkpoint blockade combined with CD19/CD22-targeted CAR-T therapy | Reviewed clinical data of CAR-T with or without allo-HSCT | N/A | Summarized overall response rate (ORR) of 70-90% for CD19 CAR-T in R/R B-ALL; combined immune checkpoint blockade (ICB) and CAR-T showed enhanced anti-tumor efficacy in preclinical and early clinical studies | No increased incidence of severe adverse events with combined ICB and CAR-T in early clinical studies | 5 |
| 6 | Wherry | 2015 | Narrative review | N/A | Reviewed molecular mechanisms of T cell exhaustion, including PD-1/PD-L1 pathway-mediated immune suppression | Not applicable | N/A | PD-1/PD-L1 blockade reverses T cell exhaustion, restores T cell effector function, and enhances anti-tumor immunity | Summarized immune-related adverse events associated with systemic ICB therapy | 6 |
| 7 | Binnewies | 2018 | Narrative review | N/A | Reviewed the role of tumor immune microenvironment (TIME) in CAR-T therapy resistance, including PD-L1-mediated immune suppression | Not applicable | N/A | Targeting TIME components (including PD-1/PD-L1 pathway) enhances CAR-T cell infiltration, persistence, and anti-tumor efficacy | N/A | 7 |
| 8 | Zhang | 2020 | Retrospective cohort study | 52 | CD19 or CD22-targeted CAR-T | All patients underwent allo-HSCT after CAR-T-induced CR | 11.1 months | 1-year OS rate of 87.7%; 1-year EFS rate of 73.0%; 1-year relapse rate of 24.7% | Grade II-IV acute graft-versus-host disease (aGVHD) in 23.1% of patients; grade III-IV aGVHD in 5.8% of patients; no treatment-related mortality | 8 |
| 9 | Zhao | 2021 | Retrospective cohort study | 105 | CD19-targeted CAR-T | 27 patients underwent allo-HSCT after CAR-T-induced CR; 78 patients underwent allo-HSCT after chemotherapy-induced CR | 49 months | 4-year OS rate of 70.2% in CAR-T-allo-HSCT group; 4-year leukemia-free survival (LFS) rate of 70.2% in CAR-T-allo-HSCT group; comparable to chemotherapy-allo-HSCT group | Grade II-IV aGVHD in 48.1% of CAR-T-allo-HSCT patients; grade III-IV aGVHD in 11.1% of patients; no difference in non-relapse mortality (NRM) between groups | 9 |
| 10 | Li | 2023 | Prospective single-arm clinical trial | 5 | Co-infusion of CD19 and CD22 dual-targeted CAR-T (1:1 ratio) | 3 patients underwent allo-HSCT after CAR-T | 26.3 months | 100% of patients achieved minimal residual disease (MRD)-negative CR; 6- and 12-month OS rate of 100% | Only grade 1-2 CRS observed; no immune effector cell-associated neurotoxicity syndrome (ICANS) reported | 10 |
| 11 | Zhang | 2024 | Prospective single-arm clinical trial | 16 | CD19/22 dual-targeted CAR-T combined with anti-PD-1 antibody tislelizumab | Not specified | 16.0 months | ORR of 87.5%; CR rate of 68.8%; 1-year progression-free survival (PFS) rate of 68.8%; 1-year OS rate of 81.3% | Grade 1-2 CRS in 50% of patients; no ICANS reported | 11 |
| 12 | Zhang | 2024 | Retrospective cohort study | 32 | Donor-derived CD19-targeted CAR-T | All patients had prior allo-HSCT and received CAR-T for post-transplant relapse | 42 months | 2-year OS rate of 56.25%; 5-year OS rate of 53.13%; 5-year EFS rate of 46.88% | No new long-term adverse events observed; manageable CRS and aGVHD | 12 |
| 13 | Luo | 2024 | Prospective phase 1 clinical trial | 9 | Donor-derived CD19-targeted CAR-T (GC007g) | All patients had prior allo-HSCT and received CAR-T for post-transplant relapse | 15.7 months | 100% of patients achieved MRD-negative CR/CR with incomplete hematologic recovery (CRi) at day 28; 1-year PFS rate of 77.8%; 1-year OS rate of 85.7% | Grade 1-4 CRS in 100% of patients; grade 1-4 aGVHD in 33.3% of patients; 1 dose-limiting toxicity observed | 13 |
| 14 | Tan | 2025 | Multicenter retrospective cohort study | 44 | CD19 Fast-CAR-T vs conventional CD19 CAR-T | Subset of patients underwent allo-HSCT after CAR-T | Not specified | CR rate of 95.7% in Fast-CAR-T group vs 71.4% in conventional CAR-T group; comparable long-term OS and LFS between groups | Higher incidence of CRS in Fast-CAR-T group (91.3% vs 66.7%); no treatment-related deaths in either group | 14 |
| 15 | Qian | 2025 | Prospective phase 2 clinical trial | 35 | Sequential CD22/CD19 dual-targeted CAR-T | Combined with autologous HSCT (sandwich strategy) | 28 months | 2-year OS rate of 97%; 2-year LFS rate of 72%; 100% of patients who completed the strategy survived | No ICANS or severe CRS observed | 15 |
| 16 | Yang | 2025 | Retrospective cohort study | 51 | CD19/CD22-targeted CAR-T | All patients underwent allo-HSCT after CAR-T-induced MRD-negative CR (88.2% haploidentical-HSCT, 11.8% unrelated/related matched HSCT) | 43.2 months | 4-year OS rate of 68.9%, 4-year LFS rate of 61.4%, 4-year cumulative incidence of relapse (CIR) of 28.0%, 4-year NRM of 10.6%; durable remissions observed in patients achieving MRD-negative CR | 100-day cumulative incidence of grades I-IV aGVHD 31.4%, grade II-IV aGVHD 15.7%; 4-year cumulative incidence of cGVHD 48.3%; no GVHD-related deaths; manageable safety profile | 16 |
| 17 | Liu | 2024 | Retrospective cohort study | 42 | CD19-targeted CAR-T | 20 patients underwent allo-HSCT after CAR-T-induced CR, 22 patients received CAR-T monotherapy | Not specified | 1-year OS rate of 70% and 1-year LFS rate of 95% in the CAR-T bridging allo-HSCT group; patients who received CAR-T bridging to allo-HSCT had higher OS and LFS rates compared with those who received CAR-T therapy alone | Comparable transplant-related toxicities between the bridging group and non-bridging group | 17 |
| 18 | Tan | 2023 | Retrospective cohort study | 43 | Donor-derived CD19-targeted CAR-T vs chemotherapy plus donor lymphocyte infusion (chemo-DLI) | All patients had prior allo-HSCT and received treatment for post-transplant relapse | Not specified | CR rate of 77.3% in CAR-T group vs 38.1% in chemo-DLI group; 2-year OS rate of 54.5% in CAR-T group vs 9.5% in chemo-DLI group | Grade 2-4 aGVHD in 9.1% of CAR-T group vs 28.6% of chemo-DLI group; manageable CRS in CAR-T group | 18 |
| 19 | Li | 2025 | Case report | 1 | CD19-targeted CAR-T derived from umbilical cord blood transplantation (UCBT) recipient | Patient had prior UCBT and received CAR-T for post-transplant relapse | 6+ years | Sustained MRD-negative CR for over 6 years; complete hematologic and molecular remission | Only grade 1 CRS observed; no GVHD or neurotoxicity reported | 19 |
| 20 | Zhang | 2022 | Retrospective cohort study | 254 | CD19-targeted CAR-T | Subset of patients underwent allo-HSCT after CAR-T | Not specified | Patients with consolidative allo-HSCT had superior OS and LFS compared with those without allo-HSCT; benefit observed in both pediatric and adult patients | Severe CRS and neurotoxicity were associated with inferior survival outcomes | 20 |
| 21 | This study | 2026 | Single-center retrospective case report | 1 | Co-infusion of CD19 CAR-T (CD28 transmembrane domain, 4-1BB costimulatory domain) and PD-L1-armored CD22 CAR-T (CD8 transmembrane domain, 4-1BB costimulatory domain, membrane-tethered anti-PD-L1 scFv) | Yes, HLA-haploidentical allo-HSCT with adjuvant umbilical cord blood mononuclear cell infusion was performed after CAR-T-induced MRD-negative CR | 7 years (84 months) | MRD-negative CR achieved on day 14 post-infusion; sustained MRD-negative remission for 7 years; 7-year EFS and OS were achieved; complete immune reconstitution by 24 months post-transplantation | Only grade 1 CRS observed, no ICANS; no acute or chronic GVHD during 7-year follow-up; no late treatment-related toxicities | - |

**REFERENCES**

1. Maude SL, Frey N, Shaw PA, Aplenc R, Barrett DM, Bunin NJ, et al. Chimeric antigen receptor T cells for sustained remissions in leukemia. N Engl J Med. 2014;371(16):1507-1517. doi:10.1056/NEJMoa1407222
2. Rafiq S, Yeku OO, Jackson HJ, Purdon TJ, van Leeuwen DG, Drakes DJ, et al. Targeted delivery of a PD-1-blocking scFv by CAR-T cells enhances anti-tumor efficacy in vivo. Nat Biotechnol. 2018;36(9):847-856. doi:10.1038/nbt.4195
3. Park JH, Rivière I, Gonen M, Wang X, Sénéchal B, Curran KJ, et al. Long-term follow-up of CD19 CAR therapy in acute lymphoblastic leukemia. N Engl J Med. 2018;378(5):449-459. doi:10.1056/NEJMoa1709919
4. Fry TJ, Shah NN, Orentas RJ, Stetler-Stevenson M, Yuan CM, Ramakrishna S, et al. CD22-targeted CAR T cells induce remission in B-ALL that is naive or resistant to CD19-targeted CAR immunotherapy. Nat Med. 2018;24(1):20-28. doi:10.1038/nm.4441
5. Wang H, Kaur G, Sankin AI, Chen FX, Guan FX, Zang XX. Immune checkpoint blockade and CAR-T cell therapy in hematologic malignancies. J Hematol Oncol. 2019;12:59. doi:10.1186/s13045-019-0746-1
6. Wherry EJ, Kurachi M. Molecular and cellular insights into T cell exhaustion. Nat Rev Immunol. 2015;15(8):486-499. doi:10.1038/nri3862
7. Binnewies M, Roberts EW, Kersten K, Chan V, Fearon DF, Merad M, et al. Understanding the tumor immune microenvironment (TIME) for effective therapy. Nat Med. 2018;24(5):541-550. doi:10.1038/s41591-018-0014-x
8. Zhang Y, Chen HR, Song YZ, Tan XY, Zhao YQ, Liu XD, et al. Post-chimeric antigen receptor T-cell therapy haematopoietic stem cell transplantation for 52 cases with refractory/relapsed B-cell acute lymphoblastic leukaemia. Br J Haematol. 2020;189(1):146-152. doi:10.1111/bjh.16339
9. Zhao YL, Liu DY, Sun RJ, Zhang JP, Zhou JR, Wei ZJ, et al. Integrating CAR T-Cell therapy and transplantation: comparisons of safety and long-term efficacy of allogeneic hematopoietic stem cell transplantation after CAR T-Cell or chemotherapy-based complete remission in B-cell acute lymphoblastic leukemia. Front Immunol. 2021;12:605766. doi:10.3389/fimmu.2021.605766
10. Li WJ, Ding LX, Shi WH, Wan XY, Yang XM, Yang J, et al. Safety and efficacy of co-administration of CD19 and CD22 CAR-T cells in children with B-ALL relapse after CD19 CAR-T therapy. J Transl Med. 2023;21(1):213. doi:10.1186/s12967-023-04019-4
11. Zhang Y, Geng HZ, Zeng LY, Li JQ, Yang Q, Jia SX, et al. Tislelizumab augment the efficacy of CD19/22 dual-targeted chimeric antigen receptor T cell in advanced stage relapsed or refractory B-cell non-Hodgkin lymphoma. Hematol Oncol. 2024;42(1):e3227. doi:10.1002/hon.3227
12. Zhang C, Wang XQ, Yi H, Wang Y, Yan ZL, Zhou J, et al. Long-term survival with donor CD19 CAR-T cell treatment for relapsed patients after allogeneic hematopoietic stem cell transplantation. J Hematol Oncol. 2024;17(1):103. doi:10.1186/s13045-024-01626-6
13. Luo Y, Gao L, Liu J, Yang LX, Wang L, Lai XY, et al. Donor-derived anti-CD19 CAR T cells GC007g for relapsed or refractory B-cell acute lymphoblastic leukemia after allogeneic HSCT: a phase 1 trial. EClinicalMedicine. 2024;67:102377. doi:10.1016/j.eclinm.2023.102377
14. Tan X, Wang JS, Chen SJ, Liu L, Li YH, Tu SF, et al. Novel CD19 Fast-CAR-T cells vs. CD19 conventional CAR-T cells for the treatment of relapsed/refractory CD19-positive B-cell acute lymphoblastic leukemia. Chin Med J. 2025;138(19):2491-2497. doi:10.1097/CM9.0000000000003479
15. Qian CS, Wang ZH, Li Z, Yao Z, Gong WJ, Gong WJ, Wu YJ, et al. A phase 2 trial of a "sandwich" strategy: sequential CD22/CD19 chimeric antigen receptor T-cells therapy combined with autologous hematopoietic stem cell transplantation in patients with Philadelphia chromosome-negative B-cell acute lymphoblastic leukemia. Cancer. 2025;131(22):e70168. doi:10.1002/cncr.70168
16. Yang TT, Dong YT, Shi JM, Zhang MM, Kong DL, Feng JJ, et al. Sequential allogeneic HSCT after CAR-T therapy for relapsed/refractory acute lymphoblastic leukemia patients: a long-term follow-up result. J Adv Res. 2025;78:461-469. doi:10.1016/j.jare.2025.02.006
17. Liu J, Xu MY, Zhang XQ, Zhang Z, Zhong T, Yu HJ, et al. CD19 chimeric antigen receptor-T cells as bridging therapy to allogeneic hematopoietic cell transplantation improves outcome in patients with refractory/relapsed B-cell acute lymphoblastic leukemia. Heliyon. 2024;10(13):e33937. doi:10.1016/j.heliyon.2024.e33937
18. Tan X, Wang XQ, Zhang C, Zhao XL, Yao H, Chen G, et al. Donor-derived CD19 CAR-T cells versus chemotherapy plus donor lymphocyte infusion for treatment of recurrent CD19-positive B-ALL after allogeneic hematopoietic stem cell transplantation. Curr Med Sci. 2023;43(4):733-740. doi:10.1007/s11596-023-2746-1
19. Li HB, Liu J, Guo D, Zhao YQ, Li Q, Qi S, et al. Case report: CD19 CAR-T cells derived from recipient of umbilical cord blood transplantation effectively treated relapsed acute lymphoblastic leukemia after UCBT. Front Immunol. 2025;16:1586349. doi:10.3389/fimmu.2025.1586349
20. Zhang X, Yang JF, Li JJ, Li WQ, Song D, Lu XA, et al. Factors associated with treatment response to CD19 CAR-T therapy among a large cohort of B cell acute lymphoblastic leukemia. Cancer Immunol Immunother. 2022;71(3):689-703. doi:10.1007/s00262-021-03009-z
